# Layer-by-Layer Surface Modification of Alendronate-Loaded Polyester Microparticles—Enabling Protein Immobilization

**DOI:** 10.3390/polym14224943

**Published:** 2022-11-15

**Authors:** Tomasz Urbaniak, Witold Musiał

**Affiliations:** Department of Physical Chemistry and Biophysics, Pharmaceutical Faculty, Wrocław Medical University, Borowska 211, 50-556 Wrocław, Poland

**Keywords:** layer-by-layer coating, polyester microparticles, macrophage targeting, protein surface immobilization

## Abstract

The highly inert surface of polyester micro- and nano- drug carriers is a challenging substrate for further modification. The presence of surface moieties suitable for macromolecule coupling is crucial in the development of targeted drug delivery systems. Among available methods of surface activation, those based on adsorption of charged macromolecules may be carried out in mild conditions. In this work, alendronate-loaded microcores of three polyesters: poly-ε-caprolactone (PCL), poly(l-lactide-co-ε-caprolactone) (PLA-co-PCL) and poly(lactic-co-glycolic acid) (PLGA) were coated with three polyelectrolyte shells composed of chitosan/heparin (CHIT/HEP), polyallylamine/heparin (PAH/HEP), and polyethyleneimine/heparin (PEI/HEP) via the layer-by-layer method. Subsequently, the feasibility of model protein immobilization on obtained shells was assessed. Electrokinetic potential measurements confirmed the possibility of deposition of all investigated coating variants, and a positive correlation between initial core ζ potential and intensity of charge alterations after deposition of subsequent layers was identified. PEI/HEP assembly was stable in physiological-like conditions, while PAH/HEP multilayers disassembled in presence of phosphate ions, and CHIT/HEP shell showed limited stability in pH 7.4. Fluorescence assays of fluorescein tagged lysozyme surface coupled via ethylcarbodiimide hydrochloride/N-Hydroxysuccinimide (EDC/NHS) click reaction with all shell variants indicated satisfying reaction efficiency. Poly-ε-caprolactone cores coated with CHIT/HEP tetralayer were selected as suitable for model IgG surface immobilization. Antibodies immobilized on the shell surface exhibited a moderate degree of affinity to fluorescent IgG binding protein.

## 1. Introduction

**Figure 1:**
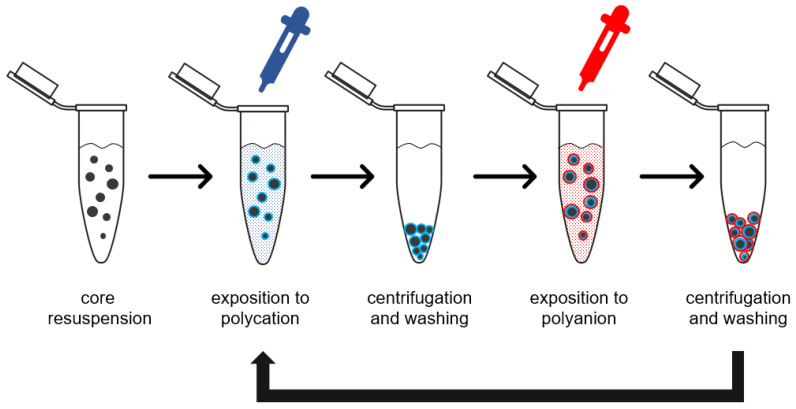
Schematic representation of layer-by-layer coating assembly on microparticle surface.

In the last three decades, biodegradable polyesters gained considerable interest from researchers focused on drug delivery systems and regenerative medicine. Due to high biocompatibility, good mechanical properties, and a variety of available monomers, they are widely applied as materials for the preparation of biomedical devices, including micro- and nano-scale drug carriers [1]. The ongoing progress in colloidal systems results in increased interest in the interaction between carrier and organism on a cellular level. The factors determining the distribution of carrier after parenteral administration are primarily particle morphology and its surface physicochemical properties. Particle size and shape determine the route of carrier elimination and patterns of organ accumulation [2]. In terms of cellular uptake, the pathway of endocytosis is also dependent on particle diameter [3]. Proper choice of particle formulation method and preparation parameters provide good control over particle size, shape, and to some extent surface morphology. However, the moieties present on the particle surface, and as a result surface charge observed on a particle, depends on the chemistry of the polymer. In the case of polyester-based particles, due to high polymer molecular weight, the presence of only a few carboxyl and hydroxyl end groups on the particle surface results in high lipophilicity and low surface negative charge in aqueous media [4,5]. Two main consequences of these properties are a tendency to aggregate and a lack of surface groups for further particle modification. The first one can be resolved by the introduction of surface-active stabilizers, which introduce repulsive forces increasing colloidal stability. The problem of lack of reactive surface groups for bioactive protein coupling is more challenging, and several techniques may be applied to address this issue. The most straightforward way to introduce a surface charge on polyester is a chemical etching with substances inducing ester bond cleavage. Polyester scaffolds soaked in sodium hydroxide exhibited significantly higher hydrophilicity in comparison to non-treated scaffolds, which led to improved cell attachment and viability [6]. The analogous effect was obtained by exposition of polyester surface to high-energy plasma, which is feasible only in the case of macro-objects [7]. Nevertheless, carboxylic and hydroxyl groups obtained in such procedures are poorly available for commonly employed protein coupling agents. To utilize surface groups obtained in mentioned procedures, macromolecular spacers have to be introduced to provide a satisfying immobilization degree and to maintain immobilized macromolecule activity [8]. Another strategy is surface grafting of polymeric chains on the polyester surface, which can be subsequently coupled with enzymes or targeting ligands [9]. Mentioned approaches require the employment of aggressive conditions or prolonged procedures, which may have a negative impact on drug incorporated in particles. More mild methods of surface modification are based on adsorption phenomena. A wide range of polyelectrolytes, including proteins, polysaccharides, and synthetic polymers, adhere to polyester surfaces via weak electrostatic forces, hydrogen bonding, and hydrophobic interactions [10]. This phenomenon is fundamental to the layer-by-layer (Lbl) surface modification method employed to assemble surface drug reservoirs, antifouling, and antibacterial coatings or to modify the interaction between biomedical devices and tissues [11]. Alternately deposited positively and negatively charged polyelectrolytes may form shells of nanometric thickness on the surface of micro- and nano- cores (Figure 1).

To achieve Lbl multilayer stability in physiological conditions, the assembly procedure is usually performed in physiological pH and ionic strength, however, additional covalent crosslinking may be employed to increase coating stability [12]. A variety of available biocompatible polycations and polyanions may be deposited on the surface of any curvature to introduce surface amine, carboxyl, hydroxyl moieties for protein coupling. Furthermore, polymer chain mobility observed in Lbl assemblies provides steric freedom [13,14], which may be beneficial in terms of protein immobilization and maintaining protein bioactivity.

Interaction of Lbl film with cells and plasma components may be influenced by the chemistry of the outer layer of assembly. It was shown that the degree and selectivity of surface cell adhesion may be controlled by modification of both Lbl film composition and assembly conditions [15]. Additionally, the chemistry of the Lbl shell deposited on particles proved to be a factor impacting the degree of internalization by phagocytic cells—macrophages [16]. Recent insights in macrophage-related pathologies revealed a number of cellular subpopulations linked to various diseases [17,18]. Multiple studies point to macrophage subtypes as critical factors in fibrosis development [19]. Other important studies indicate macrophage subpopulation involvement in the development of obesity [20]. To address the excessive activity of macrophages, alendronate (AA) may be employed to induce macrophage apoptosis. Direct inhalation and delivery via liposomes and polymeric particles were employed to reduce lung fibrosis development [21,22,23]. However, the lack of selectivity of this approach results in complete phagocytic cell eradication, which may lead to serious side effects. Drug delivery systems capable of selective elimination of particular macrophage subpopulations via membrane receptor targeting would be promising tools for addressing a variety of serious conditions.

**Figure 2:**
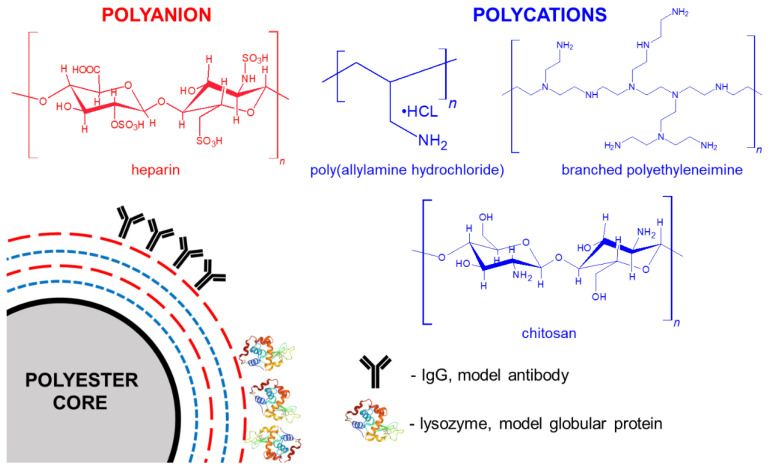
Schematic representation of fabricated core-shell microparticles with molecular structure of macromolecules employed in Lbl assembly.

The study aimed to investigate and select the variant of Lbl shell deposited on AA-loaded polyester cores suitable for surface protein immobilization. Deposition of three variants of Lbl shells composed of (1) polyethylene imine (PEI) and heparin (HEP), (2) chitosan (CHIT) and HEP, (3) polyallylamine hydrochloride (PAH) and HEP on surface of AA-loaded microparticles of three different biodegradable polyesters: polycaprolactone (PCL), poly(l-lactide-co-ε-caprolactone) (PLA-co-PCL) and poly(lactic-co-glycolic acid) (PLGA) was investigated (Figure 2).

PCL-based core-shell particles were further evaluated in terms of the shell properties and surface immobilization of proteins via a well-established carbodiimide coupling reaction [24]. Presented results provide insight in the process of Lbl film deposition and utilization of introduced surface chemical groups in targeting ligand immobilization. Preservation of coupled protein affinity indicates the possibility of cell-particle interaction modulation. The flexibility of the described system enables adaptation to different therapeutic targets, with emphasis on various disease-linked macrophage subpopulations, including alveolar macrophages to which the micro-sized carriers can be delivered by inhalation.

## 2. Materials and Methods

### 2.1. Reagents and Materials

ε-caprolactone (97%, Sigma Aldrich, St. Louis, MO, USA), DL-lactide (99%, Sigma Aldrich, St. Louis, MO, USA), tin (II) ethylhexanoate (92.5–100.0%, Sigma Aldrich, St. Louis, MO, USA), benzyl alcohol, (≥99.0%, Aldrich, Darmstadt, Germany), calcium hydride (95%, Aldrich, Germany), ethyl acetate (≥99.5%, Chempur, Piekary Slaskie, Poland), PLGA (50:50 lactide glycolide, Mw 5.000–10.000 kDa, Acros Organics, Geel, Belgium), alendronate sodium salt trihydrate (≥99.0%, Pol-Aura, Poland), sodium chloride (99.9%, Chempur, Poland), poly(vinyl alcohol) (PVA) (Mowiol^®^ 4-88, Mw~31.000, Aldrich, Germany), phosphate buffer saline (Lonza, Walkersville, MD, USA), acetic acid (96%, Chempur, Poland), chitosan (Mw~200.000, Pol-Aura, Olsztyn, Poland), polyethyleneimine branched (Mw~25.000, Aldrich, Germany), polyallylamine hydrochloride (Mw~50.000, Aldrich, Germany), heparin sodium salt (Pol-Aura, Poland), glutaraldehyde (50%, Chempur, Poland), chloroform-D (99.8%, Aldrich, Germany), tetrahydrofuran HPLC (99,8%, Chempur, Poland), polystyrene GPC standards (Sulpeco, Bellefonte, PA, USA), acetonitrile HPLC (99,9%, Chempur, Poland), tetrabutylammonium perchlorate (≥98.0%, Pol-Aura, Poland), 2-Mercaptoethanol (99%, Acros Organics, Belgium), o-phthalaldehyde (≥97.5%, Pol-Aura, Poland), sodium hydroxide (98,8%, Chempur, Poland), hydrochloric acid (37%, Chempur, Poland), fluorescein isothiocyanate (90%, Merck, Darmstadt, Germany), lysozyme, from chicken egg white (Pol-aura, Poland), normal mouse IgG (Santa Cruz Biotechnologies, Dallas, TX, USA), FITC-tagged m-IgGκ binding protein (Santa Cruz Biotechnologies, TX, USA), N-(3-Dimethylaminopropyl)-N′-ethylcarbodiimide hydrochloride (≥98%, Aldrich, Germany), N-Hydroxysuccinimide (≥98%, Aldrich, Germany), 4-morpholineethanesulfonic acid (≥99.0%, Pol-Aura, Poland), Tris buffer 1 M (Chempur, Poland), carbonate buffer 0.5 M (Alfa Aesar, Haverhill, MA, USA)

### 2.2. Methods

#### 2.2.1. PCL and PLA-co-PCL Synthesis Via Ring-Opening Polymerization

Low molecular weight PCL homopolymer and PLA-co-PCL copolymer were synthesized via ring-opening polymerization catalyzed by tin (II) ethylhexanoate and initiated by benzyl alcohol. E-caprolactone was dried over calcium hydride and distilled under reduced pressure, DL-Lactide was recrystallized twice from ethyl acetate. Reactions were carried out in bulk, under stirring at 130 °C for 3 h in a dry nitrogen atmosphere. The ratio between initiator and monomers in both reactions was 1:67, between catalyst and monomers in both reactions was 1:350 and the ratio of lactic acid and ε-caprolactone in PLA-co-PCL copolymer synthesis was 1:7. Obtained crude products were dissolved in dichloromethane, recrystallized from cold methanol, dried and stored in a desiccator.

#### 2.2.2. Gel Permeation Chromatography

The Mn and Mw values of synthesized polymers were determined with gel permeation chromatography. Analysis was performed with Thermo Scientific high-performance liquid chromatography set, Dionex Ultimate 3000 (Thermo Scientific, Waltham, MA, USA) equipped with Phenogel 103 Å column (Phenomenex, Torrance, CA, USA) in tetrahydrofuran at room temperature. The obtained molecular weight values relative to polystyrene standards were corrected by the coefficient of 0.56 [25].

#### 2.2.3. Proton Nuclear Magnetic Resonance

The ratio of monomers in PLA-co-PCL copolymer was assessed via HNMR on an ARX 300 MHz NMR spectrometer (Bruker, Billerica, MA, USA) in chloroform-d at 25 °C. The ratio between peak integrals of lactide monomer proton-derived multiplet at 5.10 ppm and ε-CL proton-derived triplet at 4.05 ppm were employed in calculations.

#### 2.2.4. AA-Loaded Polyester Core Preparation

Synthesized PCL, PLA-co-PCL, and commercially available PLGA were formulated into AA-loaded particles via the *w*/*o*/*w* double emulsion solvent evaporation technique. Primary emulsion was obtained by the homogenization of 2.0 mL of 20 mg/mL AA in 2% PVA, pH = 7.0 with 20 mL of 20 mg/mL polymer solution in dichloromethane with laboratory rotor-stator homogenizer X120 (Ingenieurbüro CAT, Ballrechten-Dottingen, Germany) for 15 min with a 25.000 rpm homogenization rate. The primary emulsion was immediately emulsified into 1 *w*/*v*% PVA, 1% *w*/*v*% sodium chloride solution for 5 min with a 16.000 rpm homogenization rate to obtain double *w*/*o*/*w* emulsion. The volatile organic phase was evaporated in a rotary evaporator at 35 °C under reduced pressure. Obtained particles were centrifuged and rinsed twice with deionized water.

#### 2.2.5. Drug Loading

The amount of drug incorporated into polyester cores via *w/o/w* double emulsion solvent evaporation method was assessed by quantitative analysis of AA remaining in supernatant isolated after centrifugation of particles obtained in evaporation procedure. The drug loading efficiency was calculated as a difference between the amount of drug employed in particle preparation and the amount of drug in the supernatant after incorporation of the procedure. AA concentrations were evaluated with the derivatization HPLC method developed by Al Deeb et al. [26] Briefly, 200 μL of analyte was transferred to a chromatographic vial, subsequently, 60 μL of derivatizing solution (1 mg/mL o-phthalaldehyde, 0.5% 2-mercaptoethanol in 0.05 M NaOH) was added and volume was completed to 1 mL with 0.05 M NaOH. Obtained mixture was left in room temperature for 1 h to react. A total of 10 μL of the resulting solution was injected into the HPLC system. Analysis was performed on the PRIMAIDE HPLC system (Hitachi, Tokyo, Japan) with UV-Vis detection at a single wavelength of 333 nm. Isocratic elution with 1 mL/min of mobile phase consisting of acetonitrile and phosphate buffer saline (PBS) pH 9.6 (15:85) containing 3 mg% of tertbutylammonium perchlorate was employed.

#### 2.2.6. Differential Scanning Calorimetry

The DSC curves of polymers and drug-loaded cores were obtained using a DSC 214 Polyma (Netzsch, Selb, Germany) heat flux-type calorimeter. Samples were sealed in 40-ll standard aluminum crucibles with a hole in the lid. The mass of a single sample was between 4 and 6 mg. The DSC cell was purged with a stream of high-purity nitrogen (99.999%) at a rate of 25 cm^3^ min^−1^. DSC scans of all the samples were run at a heating rate of 5 °C min−1 in the temperature range of 0–280 °C.

#### 2.2.7. Lbl Coating of Polyester Cores

Deposition of polyelectrolyte layers was carried out by resuspension of polyester cores in proper media followed by the addition of polyelectrolyte solution in the same media to a final polyelectrolyte concentration of 1 mg/mL. The weight ratio between cores and polyelectrolytes in all deposition steps was 6.66:1. Between each step, particles were centrifuged for 4 min with the relative centrifugal force of 3350 g, supernatants were discarded, particles were resuspended in dedicated media under mild ultrasonic treatment in an ultrasound bath and vortex mixing. Following media were employed for each multilayer variant: PBS pH 4.5 (pH adjusted with glacial acetic acid) for CHIT/HEP, PBS pH 7.4 for PEI/HEP, and deionized water for PAH/HEP. In every step, particles were shaken in polyelectrolyte solution for 10 min, and a 5 min rinsing step in corresponding media between each deposition step was employed. All media and polyelectrolyte solutions were filtered through 0.22 μm syringe filters prior to use. Fabricated bilayers and tetralayers were crosslinked with glutaraldehyde. Coated particles were resuspended in 1% glutaraldehyde solution of pH 5.5, incubated for 30 min, centrifuged, and washed twice with distilled water.

#### 2.2.8. Dynamic Light Scattering

Particle hydrodynamic diameters and particle surface electrokinetic potentials (ζ potential) were evaluated via dynamic light scattering measurements on a Zetasizer Nano apparatus (Malvern, Worcestshire, UK). ζ potential was measured in capillary cells, hydrodynamic diameter measurements were carried out in polystyrene cuvettes. Presented values were calculated from the Smoluchowski equation by dedicated software. Each point in in situ measurements of ζ potential during Lbl deposition is a value obtained after one-minute data collection. Hydrodynamic diameters and ζ potential values in equilibrium states were measured at least three times; presented values are expressed as mean ± SD.

#### 2.2.9. Scanning Electron Microscopy

The morphology of microspheres was evaluated with scanning electron microscopy. Samples were sputtered with 10 nm carbon layer and observed in a Quanta 650 microscope (15 kV, FEI, Hillsboro, OR, USA).

#### 2.2.10. Determination of Surface Carboxylic Group Content Via Conductometric Titration

Weighted core-shell particles were resuspended in 50 mL of deionized degassed water, and the pH of the suspension was increased to approximately 10.00 by the addition of 5 μL of degassed 5 M NaOH, to deprotonate all available acidic groups. Subsequently, the solution was titrated with 0.5 mM HCL with an automatic dosing system Titronic 500 (SI Analytics, Mainz, Germany) under constant stirring with nitrogen flowing through suspension. Conductivity was measured during the titration procedure with digital conductometer (Elmetron, Zabrze, Poland), and the volume of titrant necessary for carboxyl group titration was derived from intersection points of three linear functions fitted to titration curves, the blank measurement performed with deionized, degassed water was subtracted from each titration [27].

#### 2.2.11. Protein Surface Immobilization

##### Lysozyme (Lys) Fluorescent Tagging

Lys was coupled with FITC according to the manufacturer guide. Briefly, 10 mL of 0.6 mg/mL FITC solution in 0.1 M carbonate buffer was added dropwise to 10 mL of 25 mg/mL lys solution in same buffer under constant stirring. Subsequently, the solution was left to react overnight at 4 °C. Obtained FITC-lysozyme (FITC-Lys) conjugate was purified by dialysis through a dialysis membrane (Spectra/Por, Replingen, Boston, MA, USA) with a cutoff 6–9 kDa against deionized water until the conductivity of external water solution remained constant. Purified FITC-Lys solution was freeze-dried (Lyovac GT2, Steris, Köln, Germany). FITC/protein molar ratio was determined by absorbance measurements, according to manufacturer protocol and was equal to 0.66.

##### Protein Coupling on Surface of Core-Shell Microparticles

Model proteins FITC-tagged Lys and IgG were covalently bound to particle surface carboxylic group via an amide bond. A widely applied coupling method employing N-ethylcarbodiimide hydrochloride (EDC) and N-Hydroxysuccinimide (NHS) was utilized. A total of 24 μL of 10 mM EDC and 240 μL of 10 mM NHS aqueous solutions were added to 1 mL of 1 mg/mL particle suspension in 4-morpholine ethanesulfonic coupling buffer pH 6.0 (MES), and shaken out for 30 min to activate surface carboxylic groups. Subsequently, particles were centrifuged and resuspended in MES twice. Activated particles were again resuspended in MES, mixed with the desired volume of protein solution in MES, and volume was completed to 1 mL and shaken for 2.5 h. To quench the reaction and remove unreacted proteins, particles were centrifuged, resuspended in 1 mL of 50 mM Tris blocking buffer, incubated overnight in 4 °C, and washed twice in 50 mM Tris blocking buffer.

##### Fluorescence Assay

Fluorescence derived from proteins coupled to particle surface was evaluated via fluorescence intensity measurements in a GloMax Discover plate reader (Promega, Madison, WI, USA). Obtained core-shell particles with surface-immobilized FITC-lys were resuspended in 50 mM Tris buffer. A total of 100 μL of suspension was transferred to a 96-well plate, fluorescence intensity was measured at an excitation wavelength 475 nm and an emission wavelength range of 500–550 nm. Lys coupling efficiency was calculated from the concentration of Lys present in supernatant obtained after centrifugation of reaction mixture. Particles with surface-immobilized IgG were incubated for 2 h in room temperature with FITC-tagged IgG-bp in 50 mM Tris buffer, centrifuged, and washed twice. A total of 100 μL of suspension was transferred to a 96-well plate, fluorescence intensity was measured, presented values are average of three measurements. Particle-derived fluorescence was also observed under the fluorescent microscope IX53 (Olympus, Tokyo, Japan) with a mercury arc lamp as the fluorescent light source.

## 3. Results and Discussion

### 3.1. Physicochemical Properties of Polyesters

In order to obtain materials for carrier formulation, similar in terms of molecular weight, and applicable for drug release, PCL and PLA-co-PCL low molecular weight polyesters were synthesized and characterized. Properties of obtained polymers and commercially available PLGA were summarized in Table 1.

Low molecular weight polyesters were employed due to long degradation time and slow drug release from high-molecular weight polymer matrices [28]. The molecular weight of synthesized polymers was close to theoretical values, which indicate good reaction control.

### 3.2. AA-Loaded Core Formulation

Hydrophilic drug incorporation into hydrophobic polymer matrices via in *w/o/w* double emulsion solvent evaporation method is a challenging task due to low compatibility of AA and polyesters. Several works investigated the impact of various factors on AA incorporation efficiency in polyester carriers [28,29,30]. In the present work, emulsion stabilizer PVA was added to both w1 and w2 aqueous phases of a double emulsion. It was proved, that PVA can stabilize *w*/*o* emulsion despite that its HLB value being typical for an *o*/*w* emulsion stabilizer [31]. Employment of common *w/o* stabilizers such as Tweens lead to plasticization of obtained particles, which cannot be resuspended after centrifugation. Supplementation of secondary aqueous phase w2 with indifferent electrolyte increased its ionic strength and limited drug diffusion from the internal w1 aqueous phase. Microparticles obtained in the *w*/*o*/*w* double emulsion solvent evaporation method were compared in terms of drug loading efficiency and physical properties (Table 2).

**Figure 3:**
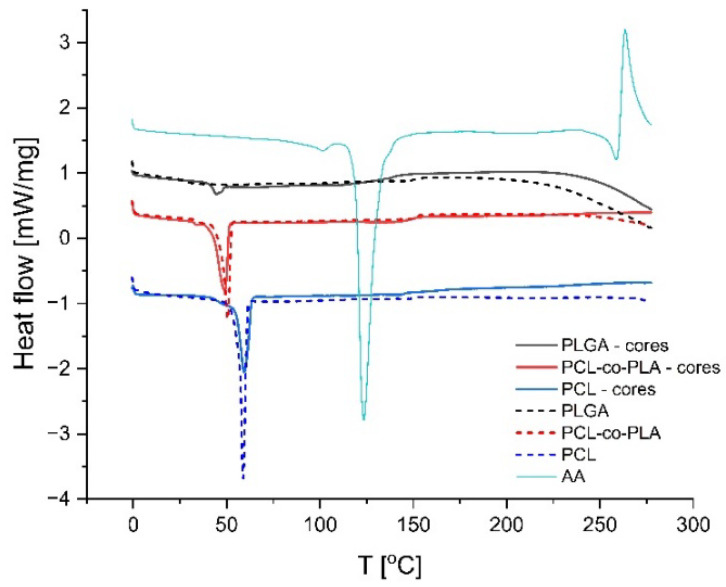
DSC curves of alendronate-loaded cores composed of PLGA, PLA-co-PCL, PCL and AA (solid lines) and pure polymers (dotted lines).

Differences in polyester hydrophilicity are crucial in terms of highly hydrophilic drug incorporation [32], which was reflected in AA loading efficiency. No drug-derived thermal peaks related to AA melting at 125 °C were observed on DSC curves of loaded particles, which indicates an amorphous form of the incorporated drug (Figure 3). For particles composed of PLGA, an endothermic peak related to glass transition was observed at 44 °C. Endothermal peaks related to melting at 48 °C and 59 °C were observed on thermograms of semicrystalline PLA-co-PCL and PCL, respectively. The observed temperature of thermal transitions did not differ from those observed for pure polymer samples and were typical for evaluated polyesters.

**Figure 4:**
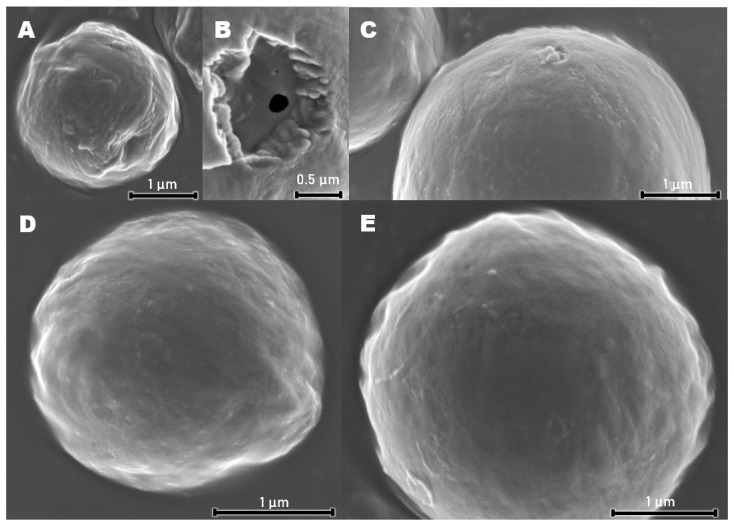
SEM micrographs of (**A**,**B**) PCL cores, and PCL cores coated with crosslinked tetralayer (**C**) PAH/HEP, (**D**) PEI/HEP and (**E**) CHIT/HEP shells.

Values of surface ζ potentials were, respectively, lower for particles formed of more hydrophilic polymers, whereas hydrodynamic diameters of all three particle batches were comparable. Morphology of PCL particles observed via SEM suggests the presence of partially interconnected pores inside particles formed during evaporation of the volatile phase (Figure 4B).

### 3.3. Layer-by-Layer Coating of AA-Loaded Cores

**Figure 5:**
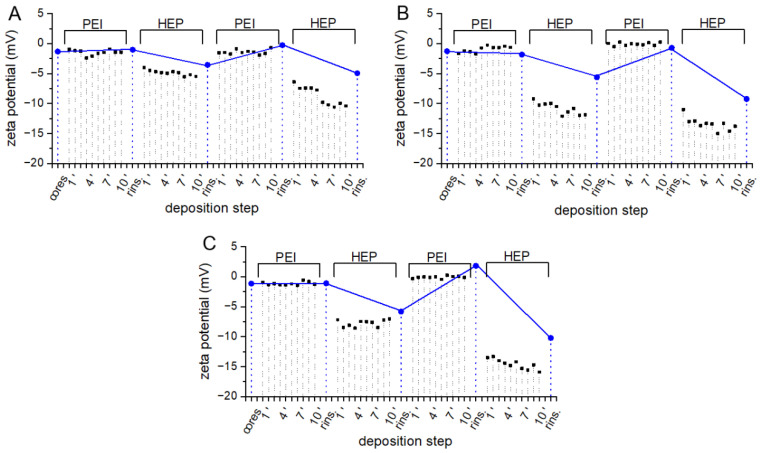
ζ potential changes in the course of PEI/HEP coating deposition in PBS pH 7.4 on (**A**) PCL, (**B**) PLA-co-PCL, and (**C**) PLGA cores. Blue dots represent ζ potential values after deposition of each monolayer and subsequent rinsing, black squares depict in situ changes in ζ potential over 10 min of exposition to polyelectrolyte solution.

Lbl coatings deposited on particle surface may provide stealth properties, stimulate mucoadhesion, increase colloidal stability or modify the kinetics of drug release. The chemistry of the shell outer layer significantly influences the mode of particle interaction with the environment, therefore numerous synthetic and natural polymers were investigated as components of Lbl assemblies [33]. In this work, deposition of three different Lbl coatings on cores fabricated of three different polyesters was investigated to select the variant most suitable for surface protein immobilization. Lbl assembly on some surfaces may be not possible due to insufficient interaction between surface and polyelectrolyte. Surface charge on polymeric materials may be introduced by chemical etching or plasma treatment. Alternatively, some macromolecules capable of strong interaction with inert surfaces e.g., polydopamine, PEI, or albumin may be employed as anchoring layers [34,35]. Due to the negative charge of polyester surfaces, three polycations: CHIT, PAH, and PEI were investigated as initial positively charged components of the film. Biocompatible polysaccharide HEP, containing both sulfate and carboxyl groups in a monomer unit, was selected as a polyanion. Strong sulfonate groups provide the necessary charge for Lbl assembly while carboxyl groups could be subsequently utilized in surface protein coupling. Due to the small total mass of polymers adsorbed in Lbl assembly procedures, concentrations of polymers employed in all experiments were considered to be excessive and sufficient for surface saturation [36]. PEI/HEP assemblies were successfully prepared on metal surfaces to reduce platelet adhesion [37,38], and on polystyrene as growth factor releasing reservoirs [39]. Lbl films designed for biomedical applications are frequently assembled in physiological-like pH and ionic strength, to obtain multilayer stability after introduction into the biological environment. The presence of phosphate ions in plasma may significantly affect the properties of polycations, including their charge and capability to interact with polyanions. Therefore, PEI/HEP coating assembly on polyester cores was carried out in PBS buffer in which PEI ζ potential was equal 0.77 ± 0,10 mV. Changes in particle ζ potential are commonly monitored during the assembly procedure as they reflect the phenomenon of surface polyelectrolyte adsorption [40]. Slight changes after the exposition of cores to PEI solution and rinsing for all three polyesters are followed by more pronounced alterations after deposition of subsequent three polyelectrolyte layers (Figure 5A–C, blue dots).

A modest increase in ζ potential, especially after the deposition of the initial PEI layer on all three core types, may be attributed to only slightly positive ζ potential observed on PEI. Ionic interactions between phosphate ions present in buffer and positively charged amine moieties of PEI result in the decreased charge of polycation molecules. The differences in ζ potential between each PEI and HEP layer are noticeably higher during deposition on more hydrophilic PLGA and PLA-co-PCL cores (Figure 5C,D, blue dots) in comparison to PCL (Figure 5A, blue dots). The density of charge-bearing moieties in employed polyesters decreases in the following way PLGA > PLA-co-PCL > PCL, which translates to a density of surface charge and capability to adsorb oppositely charged polyelectrolytes. Parallel in situ ζ potential measurements during 10 min of particle exposition to polyelectrolytes reflect changes in the composition of the electrolyte environment at the slipping plane of an electrical double layer present on the particle surface (Figure 5, black squares). Particle colloidal suspension in PEI solution exhibited an immediate slight increase in ζ potential, which did not change significantly after rinsing and resuspension in PBS. The ζ potential of microspheres with external PEI layer decreased immediately after resuspension in HEP solution, and a further moderate drop during 10-min exposition was observed. These changes in the composition of the electrical double layer suggest that accumulation of negatively charged electrolytes on particle surface occurs. Various approaches were employed to investigate the kinetics of protein and polymer adsorption on particles [41,42]. Macromolecule physicochemical properties and particle surface morphology determine the mechanism and rate of adsorption in each case under consideration. In practice, most reported Lbl deposition protocols include adsorption steps of at least 10 min to ensure sufficient interaction between oppositely charged molecules. Presented data suggest that while initial adsorption of polyelectrolyte is instantaneous, some macromolecular rearrangement and ionic exchange may occur during prolonged exposition to polyelectrolyte.

**Figure 6:**
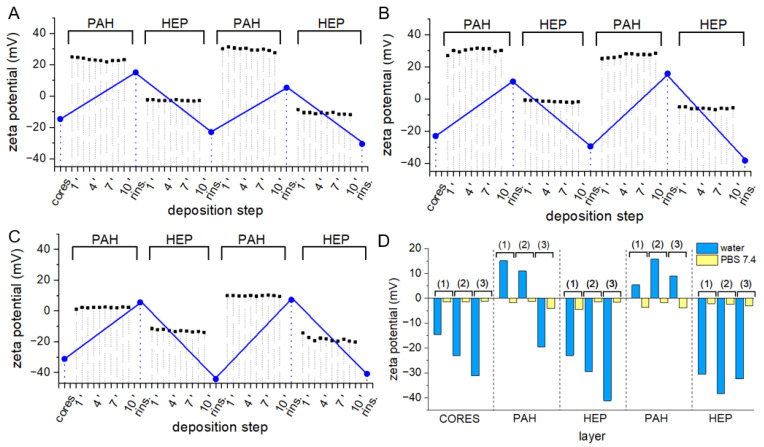
ζ potential changes in the course of PAH/HEP coating deposition in distilled water on (**A**) PCL, (**B**) PLA-co-PCL, and (**C**) PLGA cores. Blue dots represent ζ potential values after deposition of each monolayer and subsequent rinsing, black squares depict in situ changes in ζ potential over 10 min of exposition to polyelectrolyte solution; (**D**) ζ potential values of (1) PCL, (2) PLA-co-PCL, and (3) PLGA cores coated with four subsequent PAH/HEP monolayers resuspended in water (blue) and PBS 7.4 (yellow).

Second assembly variant employing PAH as polycation was reported as cell adhesion promoting and protein adsorbing surface type [43]. Due to the strong interaction between PAH and phosphate anions, [44] PAH/HEP Lbl assembly was performed in deionized water (Figure 6A–C), in which PAH ζ potential was equal 57.76 ± 1.39 mV, and multilayer stability in PBS was examined (Figure 6D)

Because of the lack of counterions in aqueous particle suspensions, observed ζ-potential values were significantly higher in the case of positively charged particles and lower in negatively charged particles. Alterations in ζ-potential typical for Lbl assembly occurred and did not differ between assemblies deposited on cores of different polyesters. Interestingly, in situ measurements indicate the stable composition of the electrical double layer on the particle surface during particle exposition to polyelectrolytes. Presumably, due to the lack of small counterions in the assembly environment, no ion-exchange reactions had to occur to establish electrostatic interaction between macromolecule [45]. To assess the stability of coatings in conditions reflecting the physiological environment, cores of all three investigated polyesters were resuspended in PBS pH 7.4 on each step of the deposition procedure. In all cases, contact with PBS led to charge reversal to values characteristic for non-coated cores (Figure 6D, yellow bars). Presumably, introduced phosphate ions exhibiting high affinity to PAH displaced HEP, and as result obtained coatings were disassembled. Due to the high plasma concentration of phosphate ions, PAH/HEP coatings exposed to physiological conditions may require prior crosslinking.

**Figure 7:**
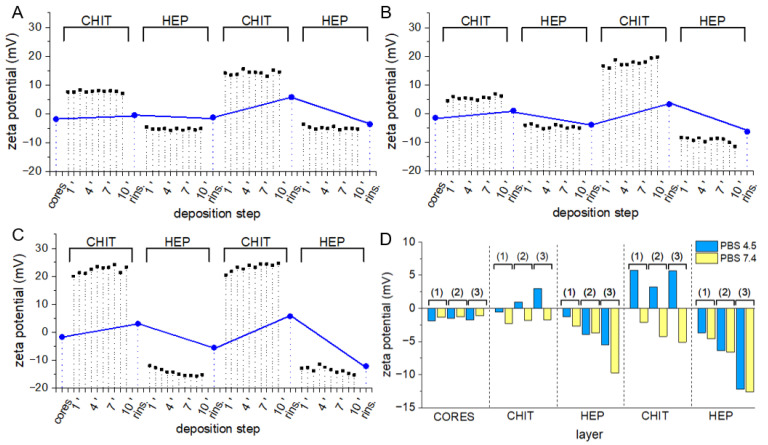
ζ potential changes in the course of CHIT/HEP coating deposition in PBS pH 4.5 on (**A**) PCL, (**B**) PLA-co-PCL, and (**C**) PLGA cores. Blue dots represent ζ potential values after deposition of each monolayer and subsequent rinsing, black squares depict in situ changes in ζ potential over 10 min of exposition to polyelectrolyte solution; (**D**) ζ potential values of (1) PCL, (2) PLA-co-PCL, and (3) PLGA cores coated with four subsequent CHIT/HEP monolayers resuspended in PBS 4.5 (blue) and PBS 7.4 (yellow).

Third coating variant employing CHIT as polycation was assembled at pH 4.5, in which CHIT ζ potential was equal PAH 23.30 ± 1.35 mV, to enable CHIT dissolution and provide the necessary charge on CHIT (Figure 7).

Similar to PEI/HEP and PAH/HEP coatings, deposition of CHIT/HEP multilayers was possible on all three polyester surfaces. In situ measurements of ζ potential during deposition of both CHIT and HEP on PLA-PCL and PLGA surfaces revealed small changes in electrical bilayer composition over 10 min of exposition (Figure 7A–C). Due to the high molecular weight of CHIT, a slower transition between different possible conformations may occur and the most favorable arrangement of macromolecules is not achieved instantaneously. Resuspension of CHIT-coated cores in PBS pH 7.4 led to a o ζ potential reversal to values observed on uncoated particles (Figure 7D). However, particles with a HEP outer layer preserved their ζ potential after resuspension in PBS 7.4. Moreover, the exposition of CHIT/HEP/CHIT coated particles to a pH of 7.4 led to a ζ potential decrease to values lower, than those observed on uncoated particles. Unlike in PAH/HEP assembly, where exposition to phosphate ions led to coating disassembly, CHIT/HEP with HEP outer layer were stable in pH 7.4. While deposition of non-modified weak acid CHIT in LbL systems is always carried out at low pH to provide a positive charge on amine groups [46], stability of CHIT/HEP in physiological pH was previously reported [47].

### 3.4. Multilayer Crosslinking

**Figure 8:**
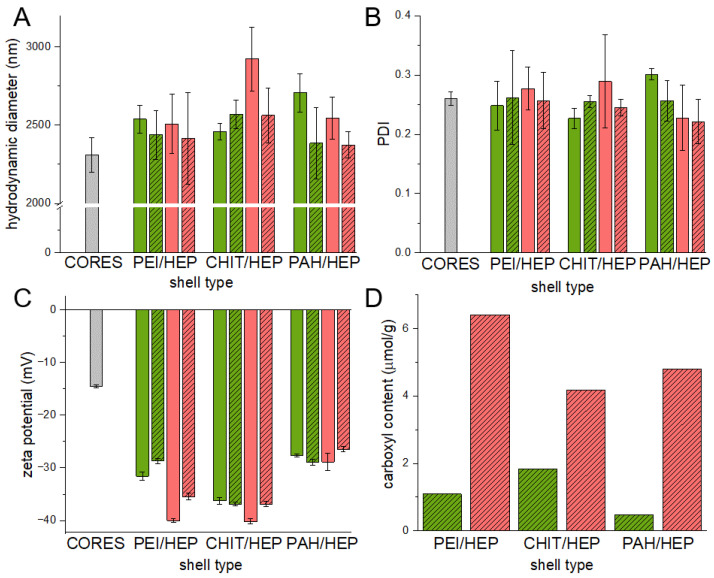
(**A**) Hydrodynamic diameter, (**B**) diameter polydispersity index, (**C**) ζ potential and (**D**) surface carboxyl content of core-shell particles with crosslinked (dashed) and non-crosslinked (plain) bilayers (green) and tetralayers (pink) of three coating variants on AA-loaded PCL cores.

PLGA and PLA-co-PCL particles are promising candidates for surface protein immobilization due to favorable loading capacity and lower values of ζ potential (Table 2), which may facilitate polycation adsorption. Nevertheless, tendency to strong aggregation after vacuum-drying or freeze-drying made their resuspension challenging. As the immobilization procedure requires monodisperse particle colloid, PCL-based core-shell microspheres were selected for model protein coupling. Covalent attachment of protein via amine group to Lbl coatings required activation of carboxyl groups on particle surface with EDC/NHS. To avoid reaction between activated carboxyl groups and amine groups present in Lbl multilayers and to increase shell stability, glutaraldehyde crosslinking of polycation with HEP amine groups was employed. Particle hydrodynamic diameter, diameter polydispersity index (PDI), and ζ potential were measured prior (Figure 8A–C plain columns) to and after crosslinking procedure (Figure 8A–C dashed columns).

In all variants of Lbl shells, hydrodynamic diameters of coated particles were larger in comparison to uncoated cores. Those differences indicate the deposition of multilayers of several hundred nanometers. No distinct features on the micrographs of three variants of tetralayer coated PCL particles were visible (Figure 4C–E). This is presumably due to the extremely low thickness of dehydrated films and the presence of a 10 nm thick layer of carbon sputtered on samples prior to the measurements. The influence of the glutaraldehyde crosslinking procedure on particle hydrodynamic diameter and PDI was negligible, indicating a lack of interparticle crosslinking and aggregation. A slight decrease in ζ potential may be attributed to side reactions of glutaraldehyde with hydroxyl groups present in Lbl multilayers [48]. The quantity of available weak acidic groups on crosslinked bilayers (Figure 8D, green columns) and tetralayers (Figure 8D, red columns) of all assemblies on PCL cores was evaluated. Measured carboxyl contents were in a range from 1–6 μmol/g depending on the coating chemistry and thickness. Although coating-derived carboxyl content was several times lower than this of commercially available functionalized polystyrene particles, it was sufficient for surface protein immobilization purposes. Significantly higher carboxyl group content in tetralayers in comparison to bilayers in all three variants was observed, which is in agreement with the reported non-linear increase in the material mass deposited via the layer-by-layer technique [10]. The abundance of carboxyl moieties on particles coated with tetralayers makes them more suitable for protein immobilization experiments.

### 3.5. Model Protein Immobilization

**Figure 9:**
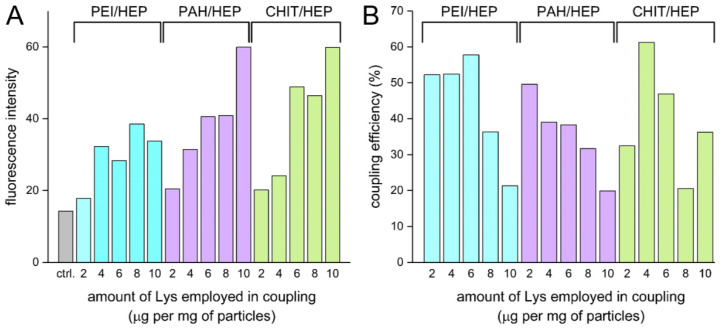
(**A**) Fluorescence intensity derived from immobilized FITC tagged Lys and (**B**) coupling efficiency of reactions carried out with different Lys concentrations.

EDC/NHS coupling strategy is considered as suitable for antibody and globular protein immobilization on surface grafted polymers or functionalized particles, with conservation of their bioactivity [49]. For covalent coupling on evaluated Lbl assemblies, model globular protein FITC-tagged Lys was selected for its structural stability. Immobilization was conducted on tetralayers of all three coating variants. Lys in the concentration range from 2 to 10 μg per mg of particles was employed. Higher protein concentrations in reaction mixtures resulted in a higher degree of protein immobilization, and considerably higher particle-derived fluorescence intensity was observed in CHIT/HEP tetralayer (Figure 9A) despite lower carboxyl group content in comparison to PAH/HEP and PEI/HEP tetralayers (Figure 8D).

**Figure 10:**
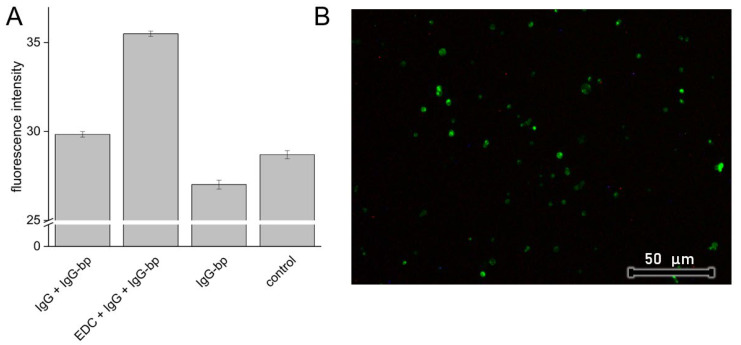
(**A**) Fluorescence intensity derived from crosslinked CHIT/HEP tetralayer coated PCL cores: subjected to EDC/NHS coupling reaction (EDC + IgG + IgG-bp), blank reaction (IgG + IgG-bp), incubated with IgG binding protein (IgG-bp) and non-treated particles (control); (**B**) Fluorescence microscopy micrograph of particles after EDC/NHS coupling reaction.

According to the proposed EDC/NHS coupling reaction mechanism, the preconcentration stage of electrostatic interaction between charged activated carboxyl group and protein amine group must occur prior to amide bond formation [50]. Presumably, due to the low CHIT pKa value, the low degree of polycation protonation provides a favorable free negative charge on HEP sulfate and carboxyl groups, and thus facilitates initial interaction with positively charged moieties of protein. At the same time, Lys surface immobilization was more efficient in reaction variants performed with a lower Lys/particle ratio (Figure 9B). Similar efficiency with an opposite relationship regarding Lys/particle ratio was reported in the corresponding procedure performed with polystyrene carboxy-functionalized particles [51]. It should be taken into consideration that due to pI values of Lys and IgG equal to 11.35 and 7.0, respectively, their net charge in the reaction environment was positive, which could facilitate non-covalent electrostatic adsorption. Such physical adsorption of antibodies on Lbl capsules was employed to significantly increase capsule affinity to human colorectal carcinoma [52]. CHIT/HEP tetralayer was selected as most promising coating variant for antibody immobilization due to most efficient Lys immobilization, observed stabilizing effect of HEP on tetralayer in pH 7.4 and confirmed biocompatibility. To examine the preservation of immobilized protein affinity, and assess the impact of electrostatic forces contribution to surface immobilization, the IgG antibody coupling procedure was carried out with the corresponding controls (Figure 10A).

Fluorescence intensity observed in PCL microparticles coated with CHI/HEP crosslinked tetralayers employed as control was comparable to this observed in the same particles incubated with FITC-labeled IgG binding protein (Figure 10A, IgG-bp) and particles subjected to blank reaction protocol, without the addition of EDC/NHS (Figure 10A, IgG + IgG-bp). 

This indicates a lack of electrostatic adsorption of IgG-bp alone and binding of IgG-bp to electrostatically adsorbed IgG. The higher intensity was observed only in particles with IgG immobilized through activation of particle surface carboxyl groups by EDC/NHS, which was also reflected in observed emission on fluorescence micrographs (Figure 10B). While this observation suggests covalent coupling of the antibody to Lbl assembly and preservation of its bioactivity, a moderate increase in fluorescence intensity suggests a low yield of the coupling reaction or a significant reduction in affinity to IgG-bp. Corresponding coupling procedures carried out with Lys resulted in satisfying coupling efficiency, which implies an appropriate choice of reaction conditions (Figure 9B). Previously reported EDC/NHS activated antibody coupling with amine groups of PAH adsorbed on gold particles resulted in strong ligand binding, probably due to larger area of nano-sized particles [53]. Lbl multilayer coatings exposed to an aqueous environment exist in a hydrated swollen state that promotes the penetration of protein into the deeper parts of the coating. This phenomenon may be exploited as a strategy of bioactive protein loading into Lbl assemblies serving as drug delivery reservoirs [54]. Due to the long step of coating exposure to IgG, antibody diffusion into the coating may have occurred, resulting in hindered selective binding of the IgG-bp.

Evaluated core-shell microparticles are candidate for targeted AA delivery system. Selective interaction between macrophage membrane receptors and immobilized antibodies may enhance phagocytic uptake by particular, disease-linked monocyte subpopulations. [55] The presented results can serve as a starting point for further studies focused on the effect of immobilized proteins on cellular uptake.

## 4. Conclusions

Alendronic acid was successfully incorporated into polyester microparticles via the *w/o/w* double emulsion solvent evaporation method, loading efficiency was positively correlated with the hydrophilicity of employed polymers. The feasibility of deposition of three Lbl shells composed of HEP as polyanion and CHIT, PAH and PEI as polycations on the surface of PCL, PLA-co-PCL, and PLGA microparticles was proven. Initial charge present on polyester cores had an impact on the assembly process, which was reflected in differences in intensity of ζ potential alterations after deposition of each monolayer. Carboxyl group content did not differ substantially between coating variants, whereas a noticeable difference was observed between bilayers and tetralayers of all assembly types. Decomposition of deposited in deionized water PAH/HEP multilayers was observed in presence of phosphate ions, while CHIT/HEP assemblies with HEP outer layer deposited in pH 4.5 were stable also in pH of 7.4. Model protein Lys and IgG coupling on PCL cores with surface modified with CHIT/HEP Lbl coating was evaluated. The efficiency of Lys immobilization from 20% to 60% was observed, while immobilized IgG showed moderate affinity to IgG binding protein.

## Figures and Tables

**Table 1:** Physicochemical properties of polyesters utilized in core formulation.

Polyester	M_calc._ [kDa]	M_n_ [kDa] ^a^	M_w_ [kDa] ^a^	PDI ^a^	Monomer ratio ^b^	Melting point [°C] ^c^
PCL	7.472 ^d^	7.373	11.923	1.66	-	58.4
PLA-co-PCL	7.453 ^d^	7.966	11.010	1.39	1:6.07	50.1
PLGA	7.500 ^e^	-	-	-	1:1	44.6

^a^ evaluated with GPC; ^b^ evaluated with HNMR; ^c^ evaluated with DSC; ^d^ Calculated from initiator: monomer ratio in reaction mixture; ^e^ Average value according to manufacturer.

**Table 2:** Selected properties of fabricated polyester cores.

Core Material	Hydrodynamic Diameter [nm] ^a^	PDI ^a^	ζ Potential [mV] ^a^	Drug Loading ^b^
PCL	2310 ± 112	0.26 ± 0.01	−14.6 ± 0.28	28% ± 3%
PLA-co-PCL	2367 ± 98	0.53 ± 0.06	−23.0 ± 0.24	39% ± 9%
PLGA	2543 ± 110	0.14 ± 0.03	−31.2 ± 0.45	90% ± 2%

^a^ determined by DLS; ^b^ determined by HPLC.

## Data Availability

Not applicable.
